# Current practice and challenges in fetal cardiac intervention

**DOI:** 10.3389/fcvm.2026.1701389

**Published:** 2026-04-02

**Authors:** Junhui Liu, Yue Wang, Yuan Gao, Gang Luo, Silin Pan

**Affiliations:** Heart Center, Women and Children’s Hospital, Qingdao University, Qingdao, China

**Keywords:** congenital heart disease, fetal cardiac intervention, fetal balloon aortic valvuloplasty, fetal atrial septal intervention, fetal pulmonary valvuloplasty

## Abstract

Fetal cardiac intervention (FCI) is a rapidly evolving technique aimed at improving outcomes in fetuses with severe congenital heart disease (CHD). Despite the significant promise of FCI, critical issues remain unresolved, including optimal intervention timing, precise selection criteria, and the management of procedure-related complications. This study reviews current clinical practices in FCI, offering a practical and informative reference for clinicians in pediatric cardiology and related fields.

## Introduction

Congenital heart disease (CHD) is one of the most common birth defects in newborns, damaging their survival and life quality. Globally, the incidence is estimated at approximately 9.4 cases per 1,000 live births (1). Fetal cardiac intervention (FCI) refers to the minimally invasive procedure performed on fetuses with severe CHDs aimed at altering the natural course of the disease and enhancing patients' survival and life quality. Since Maxwell et al. (2) reported the first case of percutaneous fetal balloon aortic valvuloplasty (FAV) in 1991, FCI has undergone continuous improvement over the past 30 years. Rates of procedural success, survival, and postnatal biventricular circulation (BVC) have shown progressive improvement, while procedure-related mortality and complications have declined (3, 4).

Given the inherent risks to both the mother and fetus, FCI is only reserved for severe, progressive, and life-threatening CHDs where meaningful clinical benefit is anticipated. Despite the therapeutic potential of FCI, critical challenges, such as optimal intervention timing, precise selection criteria, and management of procedure-related complications, remain. This review summarizes the current evidence and practices in FCI, providing clinicians with a valuable reference.

## Search strategy

This article aims to provide a narrative review of the main areas of FCI. We systematically searched the PubMed, EMBASE, and Cochrane databases from January 1990 to December 2025. The search terms include “fetal cardiac intervention,” “fetal aortic valvuloplasty,” “fetal aortic balloon valvuloplasty,” “fetal atrial septoplasty,” “fetal atrial septal stent,” “fetal pulmonary valvuloplasty,” “hypoplastic left heart syndrome,” “aortic stenosis,” “hypoplastic right heart syndrome,” “pulmonary atresia with intact ventricular septum,” “critical pulmonary stenosis,” “restrictive atrial septum,” “outcomes,” and “complications.” The included articles mainly consisted of original research, case series, and meta-analyses. We prioritized studies with larger sample sizes, multicenter data, or long-term follow-up. However, given the rarity of this field, we also included some representative small-scale case series to comprehensively present the current state of the field. The search yielded a total of 29 relevant original articles. All selected articles were subsequently incorporated into this review.

## Primary indications for fetal cardiac intervention

### Severe aortic stenosis with evolving hypoplastic left heart syndrome

Hypoplastic left heart syndrome (HLHS) is a rare congenital heart defect with an estimated incidence of 0.16–0.36 per 1,000 live births, accounting for 1.4%–3.8% of all CHDs (5). It is responsible for 23% of cardiac deaths within the first week of life (6). HLHS is characterized by underdeveloped left-sided cardiac structures that are unable to support systemic circulation (5–8), which includes left ventricle (LV) hypoplasia, hypoplastic ascending aorta, and aortic or mitral valve atresia or stenosis (9, 10). Aortic stenosis (AS) develops before 30 weeks, carries a 73% probability of progressing to HLHS (11, 12), and represents the primary target for FAV. By relieving left ventricular outflow tract (LVOT) obstruction, FAV enhances left heart outflow, reduces LV pressure, mitigates pathological remodeling and fibrosis, and improves LV development (13).

### HLHS with highly restrictive or intact atrial septum

In HLHS fetuses, interatrial communication is essential for pulmonary venous return from the left atrium (LA) to the right atrium (RA) to reduce LA pressure. Approximately 6%–19% of HLHS cases present with a highly restrictive or intact atrial septum (R/IAS) (14, 15), which elevates LA and pulmonary vein pressure, inducing pulmonary vascular, parenchymal, and lymphatic injury. Without timely intervention, most affected neonates die within hours to days after birth due to severe respiratory failure, pulmonary hypertension, or low cardiac output syndrome (16). Fetal atrial septal intervention (FASI) can restore interatrial communication to alleviate chronic pulmonary vascular remodeling (17–19).

### Pulmonary atresia or severe pulmonary stenosis with intact ventricular septum

Pulmonary atresia or severe pulmonary stenosis with intact ventricular septum (PA/IVS) with hypoplastic right heart syndrome (HRHS) is a rare and severe CHD, occurring in 4–8 per 100,000 live births and accounting for 1%–3% of all CHDs (20). Right ventricular outflow tract (RVOT) obstruction leads to elevated right ventricular (RV) pressure, ventricular hypertrophy, systolic impairment, tricuspid regurgitation (TR), and endocardial fibroelastosis (EFE), collectively impairing right heart development (21). Untreated infants with PA/IVS with HRHS have a 1-year survival of 70%–75% and a 5-year survival of 63%–67% (22). Fetal pulmonary valvuloplasty (FPV) can facilitate RV outflow, reduce RV pressure, mitigate adverse remodeling, and improve right heart development (22, 23).

## Pathophysiology and hemodynamics

### Severe aortic stenosis with evolving hypoplastic left heart syndrome

**Figure 1:**
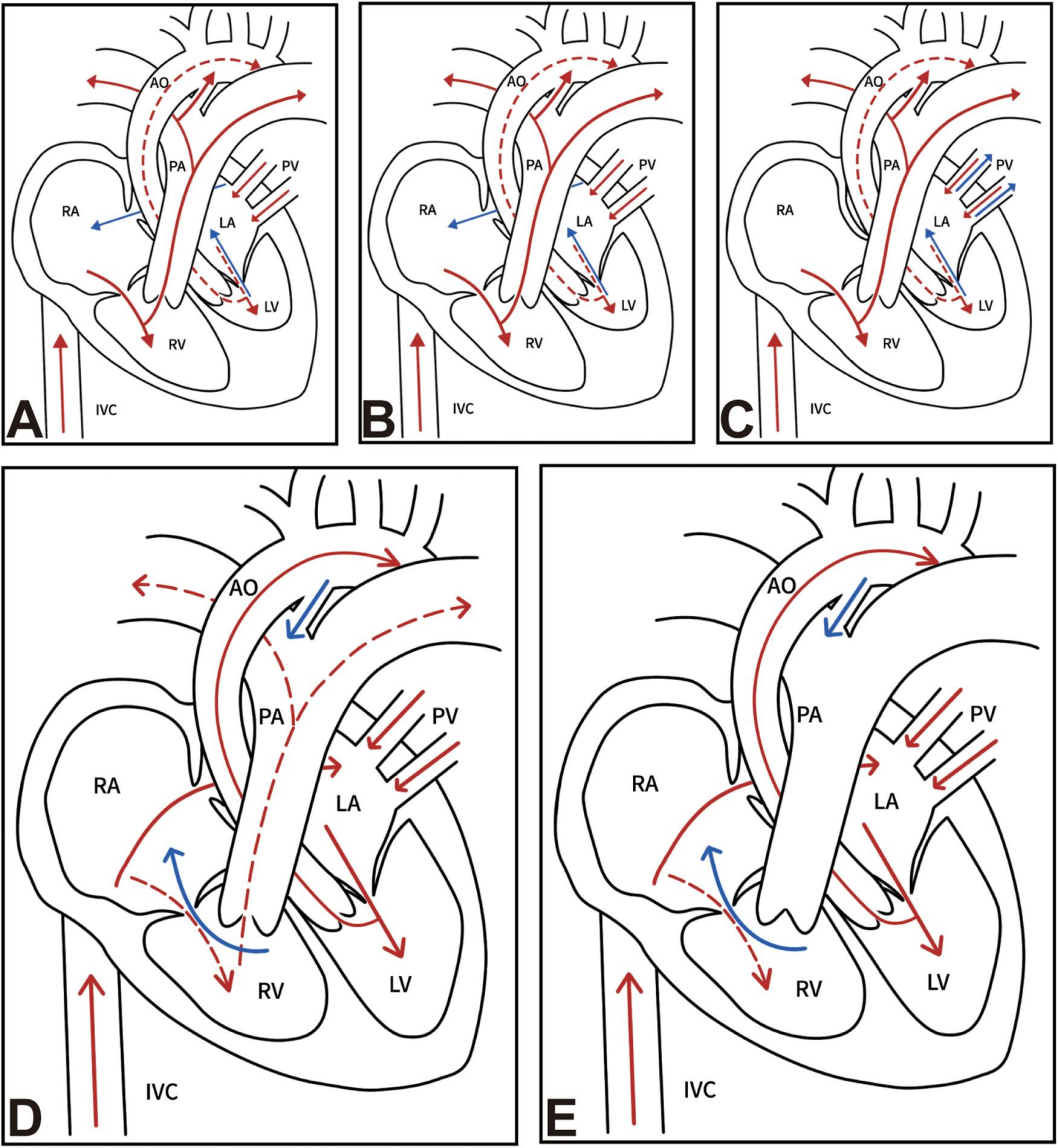
Pathophysiology and hemodynamics of congenital heart diseases. **(A)** AS with HLHS, **(B)** HLHS, **(C)** HLHS with R/IAS, **(D)** severe PS, **(E)** PA/IVS with HRHS. RV, right ventricle; LV, left ventricle; RA, right atrium; LA, left atrium; AO, aorta; PA, pulmonary artery; IVC, inferior vena cava; PV, pulmonary vein; AS, aortic stenosis; HLHS, hypoplastic left heart syndrome; R/IAS, restrictive or intact atrial septum; PS, pulmonary stenosis; PA/IVS, pulmonary atresia with intact ventricular septum; HRHS, hypoplastic right heart syndrome.

AS impairs LV outflow and elevates intracardiac pressure. This pressure overload induces LV myocardial hypertrophy and reduces chamber volume, progressively compromising left heart function. Mitral regurgitation (MR) and EFE often develop due to the pathological condition. During fetal development, the patent foramen ovale (FO) allows shunting from the LA to the RA, which mitigates LA pressure and augments pulmonary blood flow. Concurrently, the patent ductus arteriosus (PDA) permits RV output to supply the systemic circulation. Postnatally, closure of these essential shunts results in acute circulatory failure, pulmonary edema, and is typically fatal in the neonatal period without intervention (Figure 1A).

### HLHS with highly restrictive or intact atrial septum

As previously described, the patent FO in HLHS facilitates left-to-right shunting, thereby reducing LA pressure. However, in cases of HLHS with a R/IAS, this shunt pathway is compromised, leading to elevated LA pressure and impaired pulmonary venous return. These hemodynamic alterations contribute to pulmonary venous remodeling and lymphatic dilation. In such fetuses, the impaired systemic circulation is dependent on a PDA. Following birth, closure of the PDA precipitates rapid clinical deterioration, characterized by pulmonary edema, low cardiac output, and refractory hypoxemia within hours, culminating in a high early mortality rate of infants (24) (Figures 1B,C).

### Pulmonary atresia or severe pulmonary stenosis with intact ventricular septum

PA/IVS reduces RV outflow, resulting in elevated right heart pressure. Chronic pressure overload induces myocardial hypertrophy, impairs right heart function, and restricts chamber development, with severe cases progressing to HRHS. TR is frequently present unless right heart structures are diminutive. Increased RA pressure augments right-to-left shunting across the FO, leading to LA and LV dilation. Meanwhile, the PDA provides retrograde aortic flow to maintain pulmonary circulation. Moreover, sustained RV hypertension may impair coronary perfusion and promote the development of an RV-dependent coronary circulation (25). Postnatal closure of the PDA critically compromises pulmonary blood flow, culminating in severe hypoxemia and neonatal death (Figures 1D,E).

## Patient selection

### Severe aortic stenosis with evolving hypoplastic left heart syndrome

Fetuses with AS, who are at substantial risk of HLHS progression without intervention and possess sufficient potential for post-procedural LV recovery to support systemic circulation, represent the primary candidates for FAV (26). To date, recent literature has outlined patient selection criteria of FAV primarily based on the clinical experience of two leading expert centers: the Boston and Linz groups. The Boston working group proposed a combination of LV pressure >47 mmHg and ascending aorta *Z*-score ≥ 0.57 as key criteria (27). The Linz working group suggested a RV/LV length ratio <1.09 or a RV/LV length ratio of 1.09–1.3 combined with MR Vmax >314 cm/s as selection criteria (28). Recently, Green et al. (29) identified peak systolic myofiber stress as a predictor of BVC outcome after FAV, outperforming all echo parameters.

### HLHS with highly restrictive or intact atrial septum

No well-established FASI patient selection criteria exist for HLHS with R/IAS. Gellis et al. (30) reported that a ratio of pulmonary venous antegrade-to-retrograde flow velocity-time integral <2.7 predicts postnatal hemodynamic instability, urgent intervention, death, or transplantation. Additional indicators include IAS or RAS ≤1 mm, LA and pulmonary vein dilation, bidirectional pulmonary venous flow with significant systolic retrograde flow, and minimal or no early diastolic antegrade pulmonary venous flow (31). Acute maternal hyperoxygenation (AMH) testing with a cut-off value of <10% pulmonary vascular reactivity shows 100% sensitivity and 94% specificity in identifying the need for urgent postnatal intervention after FASI (32).

### Pulmonary atresia or severe pulmonary stenosis with intact ventricular septum

There were no well-established FPV selection criteria for PA/IVS. TV Z-score and qualitative assessment of RV size are commonly used to guide decisions on FCI eligibility. A tricuspid valve annulus z-score falling between −2.5 to −4 for gestational age may be used as an inclusion criterion, but it is not universally used, and RV size should be no more than moderate RV hypoplasia (33). Previous studies proposed some predictors of postnatal UVC after FPV (34–36). Wolter et al. (36) found that for fetuses at 24–30 weeks, a TV/MV < 0.62 had a sensitivity of 85% and a specificity of 88% for predicting UVC outcomes. For fetuses > 30 weeks, a TV/MV < 0.71 had a sensitivity of 91% and a specificity of 92% for predicting UVC outcomes.

## Procedural techniques

### Severe aortic stenosis with evolving hypoplastic left heart syndrome

**Figure 2:**
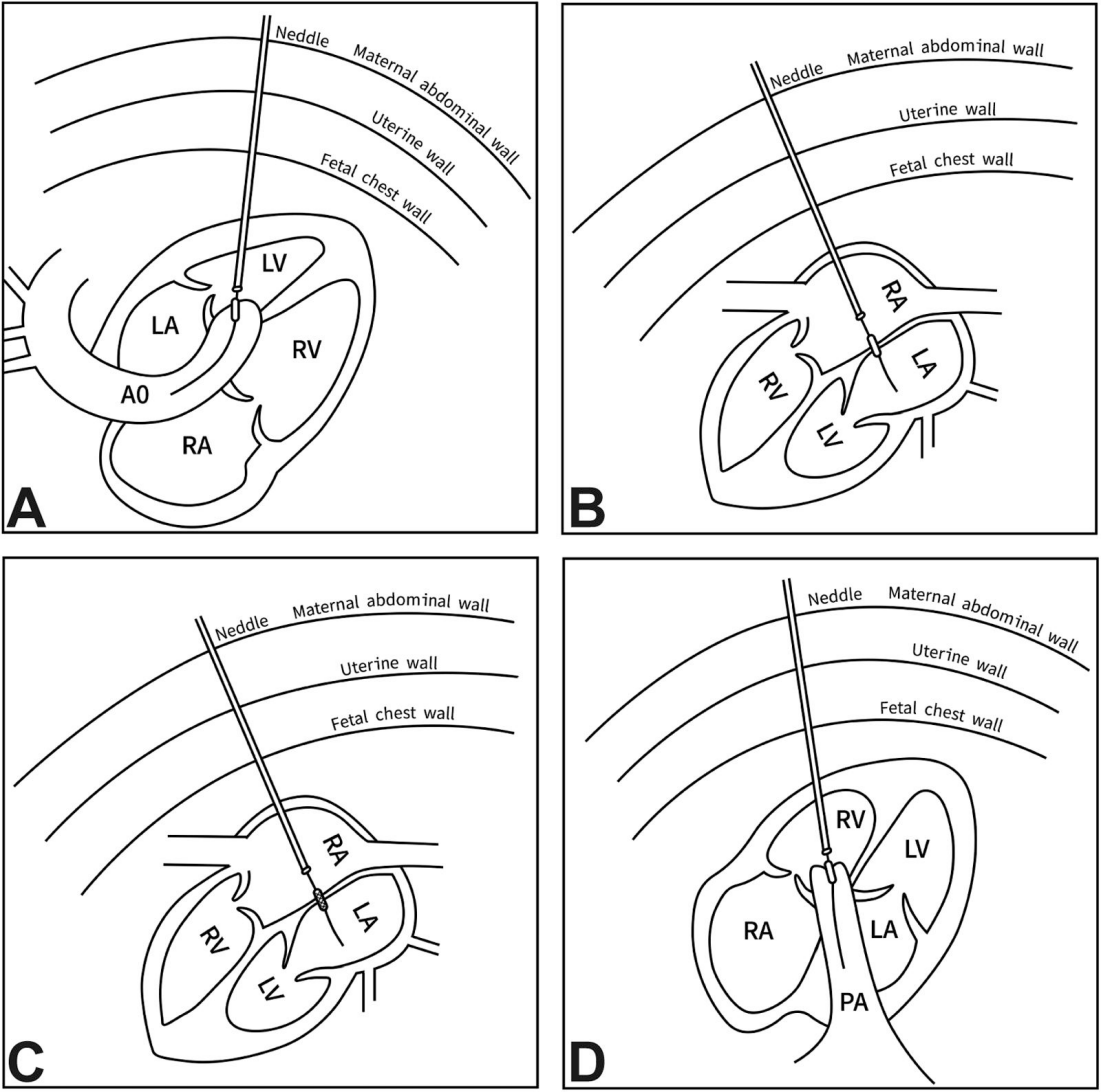
Procedural techniques of fetal cardiac intervention. **(A)** FAV is performed using an 18 G/19 G needle, a 0.014-inch coronary guide wire, and a coronary balloon. **(B)** Perforation of the atrial septum is performed using an 18 G/19 G needle, a 0.014-inch coronary guide wire, and a coronary balloon. **(C)** Stenting of the atrial septum defect with a short stent. **(D)** FPV is performed using an 18 G/19 G needle, a 0.014-inch coronary guide wire, and a coronary balloon. RV: right ventricle, LV, left ventricle; RA, right atrium; LA, left atrium; PA, pulmonary artery; AO, aorta; FAV, fetal aortic valvuloplasty; FPV, fetal pulmonary valvuloplasty.

FAV is optimally performed between 18 and 30 weeks of gestation (37). The ideal fetal position is with the fetal LV facing the maternal abdominal wall (38). An 18- or 19-gauge needle penetrates the maternal abdominal and uterine walls, entering the fetal thorax via a subcostal approach or intercostal space adjacent to the fetal sternum. The needle tip targets the LV apex, passes through the ventricular wall, and is directed toward the LVOT. A 0.014-inch coronary guidewire and angioplasty balloon are advanced through the needle. The guidewire is maneuvered across the AV into the ascending aorta, positioning the balloon across the aortic annulus. The balloon is inflated 1–2 times (39). Technical success is defined by improved antegrade flow across the AV and/or new aortic regurgitation (AR) (39, 40) (Figure 2A).

### HLHS with highly restrictive or intact atrial septum

FASI is typically performed between 24 and 34 weeks of gestation (40). The ideal fetal position is with the fetal atrium facing the maternal abdominal wall (37, 39). An 18- or 19-gauge needle penetrates the maternal abdominal and uterine walls, entering the fetal thorax via a subcostal approach or intercostal space adjacent to the fetal sternum. The needle is then inserted through the atrial wall and directed toward the atrial septum. Then the atrial septum is punctured. A 0.014-inch coronary guidewire and angioplasty balloon are advanced through the needle. The guidewire is maneuvered across the atrial septum into the other side fetal atrium, positioning the balloon across the atrial septum. Then the balloon was dilated 1–2 times (39, 40). For a thick atrial septum, stent placement is a better alternative to maintain patency. Quintero et al. (41) first used a Neodymium-YAG laser for fetal atrial septum opening, and Belfort et al. (42) reported Thulium laser-assisted fetal atrial septal stent placement. This method avoids tissue recoil and is not limited by balloon and needle size (Figures 2B,C).

### Pulmonary atresia or severe pulmonary stenosis with intact ventricular septum

FPV is optimally performed between 21 and 32 weeks of gestation (21, 22, 33, 43). The ideal fetal position is with the fetal RV facing the maternal abdominal wall. An 18- or 19-gauge needle penetrates the maternal abdominal and uterine walls, entering the fetal thorax via a subcostal approach or intercostal space adjacent to the fetal sternum. The needle tip targets the RV apex, passes through the ventricular wall, and is directed toward the RVOT. Puncture of the atretic PV is needed by introducing another ultrasharp needle. Then, a 0.014-inch coronary guidewire and angioplasty balloon are advanced through the needle, positioning the balloon across the stenotic PV. The balloon is dilated 1–2 times. Technical success is defined by improved antegrade blood flow across the PV (39, 43) (Figure 2D).

## Procedural outcomes

### Severe aortic stenosis with evolving hypoplastic left heart syndrome

Recent large cohort studies show improved FAV technical success, decreased procedural mortality, and a lower procedural complication rate for AS with HLHS. International Fetal Cardiac Interventional Registry (IFCIR) reported 81% technical success rate and 12% fetal mortality of FAV (3). Boston's cohort showed the technical success rate increasing from 73% to 94% and the fetal mortality declining to 4% (2000–2015) (27). Children's Heart Center Linz also reported improved outcomes after 2014 (28) (Table 1). Reported BVC rates after FAV range from 40% to 74% (3, 17, 22, 27, 28, 44–52) (Table 1). However, Vorisek et al. (53) found no significant difference in BVC rates between FAV and natural history cohorts. Given the lack of randomized clinical trials, these results should be interpreted with caution.

**Table 1:** Procedural outcomes of FCI in CHDs.

	Author	Cases	Technical Success % (*n*)	Procedural Mortality % (*n*)	Liveborns % (*n*)	BVC % (*n*)
Procedural outcomes of FAV in AS with HLHS						
1	Arzt et al. (45)	23	70% (16/23)	13% (3/23)	93% (15/16)	67% (10/15)
2	Freud et al. (46)	100	77% (77/100)	11% (11/100)	91% (70/77)	50% (35/70)
3	Pedra et al. (47)	13	92% (12/13)	0	100% (11/11)	46% (5/11)
4	Moon-Grady et al. (3)	86	81% (70/86)	12% (10/86)	80% (56/70)	46% (24/56)
5	Wohlmuth et al. (48)	31	94% (29/31)	7% (2/29)	79% (23/29)	61% (14/23)
6	Jaeggi et al. (49)	12	92% (11/12)	25% (3/12)	–	–
7	Galindo et al. (50)	28	79% (22/28)	29% (8/28)	50% (11/22)	55% (6/11)
8	Kovacevic et al. (51)	67	88% (59/67)	9% (6/67)	80% (47/59)	40% (19/47)
9	Friedman et al. (27)	123	82% (101/123)	9% (11/123)	92% (93/101)	45% (42/93)
10	Cruz-Lemini et al. (52)	9	100% (9/9)	33% (3/9)	67% (6/9)	67% (4/6)
11	Debska et al. (17)	94	89% (84/94)	7% (7/94)	–	–
12	Tulzer et al. (28)	103	87% (90/103)	11% (11/103)	91% (82/90)	74% (61/82)
13	Corroenne et al. (44)	58	86% (50/58)	19% (11/58)	66% (38/58)	74% (28/38)
14	Zhao et al. (22)	2	100% (2/2)	0	100% (2/2)	50% (1/2)
Procedural outcomes of FASI in HLHS with R/IAS						
1	Marshall et al. (55)	7	86% (6/7)	14% (1/7)	83% (5/6)	–
2	Marshall et al. (56)	21	90% (19/21)	10% (2/21)	100% (19/19)	–
3	Chaturvedi et al. (19)	4	100% (4/4)	0	100% (4/4)	–
4	Pedra et al. (47)	4	100% (4/4)	25% (1/4)	75% (3/4)	–
5	Herberg et al. (57)	6	100% (6/6)	83% (5/6)	0	–
6	Kalish et al. (58)	9	56% (5/9)	11% (1/9)	100% (5/5)	–
7	Moon-Grady et al. (3)	37	65% (24/37)	8% (3/37)	92% (22/24)	–
8	Jaeggi et al. (49)	2	100% (2/2)	0	100% (2/2)	–
9	Jantzen et al. (59)	47	77% (36/47)	13% (6/47)	–	–
10	Mackesy et al. (18)	31	90% (28/31)	7% (2/31)	89% (25/28)	–
11	Debska et al. (17)	19	84% (16/19)	16% (3/19)	–	–
12	Yilmaz Furtun et al. (60)	25	72% (18/25)	20% (5/25)	100% (18/18)	–
Procedural outcomes of FPV in PA/IVS						
1	Tulzer G et al. (69)	2	100% (2/2)	0	100% (2/2)	100% (2/2)
2	Montes et al. (64)	4	100% (4/4)	0	100% (4/4)	25% (1/4)
3	Pedra et al. (47)	4	75% (3/4)	0	100% (3/3)	100% (3/3)
4	Moon-Grady et al. (3)	16	69% (11/16)	13% (2/16)	64% (7/11)	71% (5/7)
5	Jaeggi et al. (49)	4	75% (3/4)	25% (1/4)	–	–
6	Tulzer et al. (62)	23	91% (21/23)	0	100% (21/21)	71% (15/21)
7	Hogan et al. (23)	58	71% (41/58)	12% (7/58)	85% (35/41)	77% (27/35)
8	Debska et al. (17)	15	80% (12/15)	7% (1/15)	–	–
9	Pang et al. (65)	5	100% (5/5)	0	100% (5/5)	60% (3/5)
10	Luo et al. (20)	7	100% (7/7)	0	71% (5/7)	100% (5/5)
11	Zhao et al. (22)	3	100% (3/3)	0	100% (3/3)	100% (3/3)
12	Deren et al. (68)	2	100% (2/2)	0	100% (2/2)	100% (2/2)

BVC, biventricular circulation. Liveborns refer to the number of liveborns in the successful FCI group. BVC refers to the number of liveborns achieving BVC with successful FCI.

This apparent discrepancy raises a critical question of whether FAV truly alters the disease course. Fetuses undergoing FAV are typically rigorously selected based on favorable LV anatomy and preserved residual LV function, which also predict a better natural history, potentially masking the true effect of the intervention (26, 27). Currently, available data do not suggest a true benefit of FAV in achieving BVC (53). A decision-analysis study further suggested that expectant management may be preferable when procedural mortality of FAV for evolving HLHS exceeds 12% or BVC rates fall below 26% in some centers (54). These findings underscore that, under current technical conditions, the potential benefit of FAV may be limited to specific patient subgroups and to experienced centers with high procedural volumes.

Common post-FAV complications include AV restenosis, progressive AR, and LV dysfunction, necessitating further interventions (25, 26, 66). A study reported 89.3% of BVC-achieving cases required reintervention, with 39.3% undergoing a second and 14.3% a third (44), which indicates the importance of long-term postoperative and postnatal follow-up.

Overall, FAV for AS with HLHS requires careful patient selection and should be performed in high-volume, experienced centers. Integrating detailed fetal echocardiographic parameters is critical for identifying suitable candidates.

### HLHS with highly restrictive or intact atrial septum

FASI technical success rate and procedural mortality for HLHS with R/IAS remain uncertain due to limited evidence. IFCIR reported 77% overall technical success rate, 13% procedural mortality, and 64% complication rate of FASI (59). Other studies report technical success rates of 56%–90% (3, 17, 18, 55, 56, 58–60), procedural mortality of 7%–83% (3, 17, 18, 47, 55–60), and live birth rates of 75%–92% (3, 18, 47, 55) (Table 1). Yilmaz Furtun et al. (60) reported improved technical success from 58% to 85% (2012–2024) due to thulium laser-assisted puncture introduction in 2018. Maintaining patency of the atrial septum is critically important for fetuses with HLHS and R/IAS. Marshall et al. found that the establishment of an atrial communication >3 mm was associated with higher postnatal oxygen saturation and improved outcomes following stage I palliative surgery (56). A meta-analysis demonstrated that the FASI group had a lower rate of postnatal restrictive atrial septum compared to the non-FASI group (67), indicating that FASI can reduce the need for emergent atrial septostomy in neonates.

Studies have indicated that stent placement is more effective than isolated balloon dilation in maintaining persistent septal patency for a thickened atrial septum (59), a finding supported by clinical data from a Polish cohort. In this cohort, 19 fetuses with HLHS and R/IAS underwent FASI. Of these, 14 received stent placement, yielding an 86% technical success rate and a 100% live birth rate. In contrast, the remaining 5 fetuses who underwent isolated balloon dilation achieved effective interatrial communication in only 1 case, which involved a thin foramen ovale flap (17). Moreover, stent placement is also highly dependent on increased experience and technological advancements. In 2014, Kalish et al. (58) reported attempted fetal atrial septal stent placement in 9 fetuses with HLHS and IAS. Successful stent placement was achieved in 4 fetuses. In the remaining cases, stent placement failed due to malposition or embolization. More recently, Yilmaz Furtun et al. (60) reported that with the addition of laser assistance and the utilization of various ultrasound imaging planes, the technical success rate of stent placement has significantly improved.

The significant heterogeneity in FASI outcomes profoundly reflects the procedure's high dependence on center experience and procedural volume (17, 18, 55–60). FCI is technically demanding, with a notably steep learning curve (63). Research has demonstrated that in ovine models, simulation-based training and repetitive practice significantly reduce the time required for needle tip positioning, suggesting that simulation is an effective means of enhancing technical proficiency (63). Similarly, centers such as Boston Children's Hospital have achieved substantial improvements in clinical outcomes as their interventional experience and procedural volume increased (60), which indicates that concentrating complex FCI in high-volume centers is essential for ensuring procedural success and safety. However, current studies on FASI generally involve small sample sizes, with the largest cohort study comprising fewer than 50 cases, which limits a robust assessment of its outcome stability.

Therefore, emerging centers planning to implement FASI should complete comprehensive simulation-based training and establish partnerships with experienced centers for technical guidance. Furthermore, future efforts should prioritize multicenter registries and collaborative studies to refine patient selection criteria and optimize technical advancements for FASI.

### Pulmonary atresia or severe pulmonary stenosis with intact ventricular septum

Procedural outcomes of FPV in PA/IVS are also uncertain due to limited evidence. IFCIR reported 71% technical success rate, 55% complication rate, and 12% procedural mortality (23). Other studies report technical success rates of 69%–91% (3, 17, 23, 47, 49, 62), procedural mortality of 7%–25% (3, 17, 23, 49), and live birth rates of 64%–85% (3, 20, 23) (Table 1). Small studies report 100% technical success and no procedural death (20, 22, 61, 64, 65, 68, 69).

Current evidence indicates that FPV can improve RV hemodynamics and promote RV growth of fetuses with PA/IVS or CPS to increase the likelihood of post-procedural BVC. Post-procedural BVC rates range from 25% to 77% (3, 23, 62, 64, 65), with 100% in some small studies (20, 22, 47, 68, 69) (Table 1). IFCIR showed fetuses with successful FCI exhibited greater increases in TV absolute measurements and Z-scores (23). Similarly, Tulzer et al. (62) and Luo et al. (20) observed significant improvements in the RV growth, confirmed by the TV/MV ratio, RV/LV ratio, and TR velocity improvements following FPV. Luo et al. (20) also found that after birth, the diameter of the TV annulus continued to improve, with significant developmental progression noted between 6 months and 1 year of age.

Post-procedural PV restenosis is common, often requiring postnatal repeat balloon dilation or surgical valvuloplasty. PDA stent implantation may also be required to maintain pulmonary circulation for subsequent staged surgical procedures in these patients (61). Although PV restenosis frequently occurs as the pregnancy progresses, FPV still promotes fetal RV growth trajectories in the early weeks (62).

Overall, the improvement in TV and RV development indicates that FPV may alter the natural course of PA/IVS in select fetuses, thereby establishing its role as a viable and promising intervention. Future research must now focus on long-term prognostic data to comprehensively assess postoperative ventricular function, quality of life, and survival.

## Complications and prognosis

### Bradycardia and pericardial effusion

Fetal bradycardia occurs in 33% of FCI cases, most frequently during FPV. Acute bradycardia, which typically occurs immediately upon cardiac puncture, is likely mediated by a vagal reflex and usually resolves spontaneously. Persistent bradycardia may require transplacental atropine administration. Refractory cases necessitate direct intracardiac epinephrine injection, with advanced resuscitation protocols indicated if bradycardia persists for more than 30 seconds after two doses (4). Progressive bradycardia is often associated with pericardial effusion. Tulzer et al. (4) recommend immediate intervention, including intracardiac epinephrine administration or effusion drainage, for bradycardia related to effusion. Effusions occurring after needle withdrawal may require a separate puncture for drainage. Asymptomatic effusions can be conservatively monitored. Debska et al. (17) advocate proactive drainage, noting that even a small volume (2–3 mL) of pericardial blood may compromise fetal cardiac function. Overall, the management of fetal bradycardia should be individualized based on institutional experience, weighing fetal benefit, procedural risks, and maternal safety.

### Aortic regurgitation and left ventricular diastolic dysfunction

Long-term follow-up demonstrates that AR and LV diastolic dysfunction are common complications following FAV, necessitating continuous surveillance. Tulzer et al. (70) reported AR in 87% of FAV recipients, with 45% experiencing significant regurgitation, which correlated with higher balloon-to-annulus ratios. Although AR often improves after birth, some cases require repeat AV interventions, such as balloon dilation or surgical valvuloplasty. Kido et al. (71) identified an aortic annulus *Z*-score <−2.6 and a history of FAV as predictors of neonatal AV intervention. Additionally, Barry et al. (72) observed progressive LV diastolic dysfunction, while Friedman et al. (73) documented universally abnormal LV diastolic parameters in FAV patients, associated with enlarged or spherical LV morphology, EFE, and lower LV pressure. Notably, EFE resection may reduce LV end-diastolic pressure (71).

### Pulmonary restenosis or re-atresia

Progressive PV restenosis or re-atresia is a frequent post-FPV complication. Luo et al. (43) observed progressive PV restenosis with increasing TR in 13 successfully treated fetuses, with one case progressing to re-atresia. Pang et al. (65) reported an 80% PV restenosis rate. Tulzer et al. (62) documented progressive PV gradients in 65.7% of cases and four cases of re-atresia, and believed a ratio of 1.3–1.5 may optimize outcomes. Managements include postnatal repeat balloon dilation or surgical valvuloplasty (62, 74).

### Neurodevelopmental and quality-of-life outcomes

Current evidence regarding long-term neurodevelopmental outcomes and quality of life in patients following FCI remains inconclusive. Normal placental blood flows through the FO into the LA, LV, and ascending AO, ultimately reaching the brain and maintaining adequate cerebral oxygenation. Different types of CHD interfere with the fetal cerebral perfusion, oxygenation, and brain metabolism (75). Chronic hypoxia and ischemia impair fetal brain development. At delivery, this may manifest as delayed brain maturation in term newborns with CHD, with brain development approximately four weeks less mature than expected (76). And fetal MRI has revealed reduced intracranial and total brain volumes, delayed gyration, and decreased surface area in both cerebral hemispheres in cases with complex CHD (77, 78). Microcephaly, holoprosencephaly, and agenesis of the corpus callosum are observed in approximately 25% of HLHS fetuses (79). Juergensen et al. (80) reported that lower fetal cardiac output correlates with reduced total brain volume. Specifically, a 10% increase in cardiac output was associated with an approximate 8 mm^3^ increase in brain volume. These findings indicate that FCI may potentially promote neurodevelopment by enhancing cerebral perfusion.

However, a 12-year follow-up study of 69 fetuses with AS who underwent FAV demonstrated no significant neurodevelopmental advantage compared to untreated patients, with both groups scoring below normative levels. Specifically, children who underwent FAV have significant impairment in their general adaptive functioning, reflecting difficulties achieving age-appropriate self-care skills and independence (81). They believed that intrinsic patient factors and infant morbidity are likely the primary determinants of long-term neurodevelopmental outcomes. This suggests that although FCI aims to optimize cardiac structure to improve cerebral hemodynamics, it may not fully reverse pre-existing brain damage caused by low cerebral perfusion or intrinsic developmental abnormalities, suggesting the potential for optimizing the timing of intervention. Studies have identified small fetal brain volume as an independent predictor of 2-year neurodevelopmental outcomes in CHD, positioning it as a key imaging biomarker for future risk (82). This underscores the importance of serial fetal brain imaging, such as MRI and ultrasonography, throughout gestation to assess the fetal brain development, which also serves as a critical reference for determining the optimal timing of FCI.

Future research should employ standardized, multidimensional neuropsychological assessment tools to conduct long-term, prospective follow-up of survivors of different types of FCI, which are crucial for optimization of interventional strategies.

## Prospects

### Innovations in imaging

Fetal echocardiography remains the primary modality for preoperative assessment and intraoperative guidance in FCI. However, its application is often limited by maternal body habitus, fetal position, and gestational age. Recently, fetal cardiovascular magnetic resonance (CMR) has emerged as a valuable complementary tool. Ryd et al. (83) reported that fetal CMR influenced clinical decision-making in 84% of cases, including delivery planning, postnatal evaluation, and interventional strategies. Nevertheless, fetal CMR is always associated with higher costs, longer acquisition times, and larger fetal size, limiting its widespread use in the early and mid-second trimester. A key direction for future imaging advancements is the establishment of standardized imaging criteria for precise preoperative selection and postoperative follow-up evaluation in FCI. Specifically, there is a need to: (1) establish multicenter collaborative networks and conduct prospective studies to validate and explore novel echocardiographic parameters, such as LV myocardial fiber stress (29) and pulmonary venous flow patterns (30) for predicting procedural outcomes; (2) explore the added value of fetal CMR or MRI in assessing myocardial fibrosis, pulmonary vascular, and brain development, and investigate its potential for evaluating the long-term prognosis of fetuses undergoing FCI (15); and (3) promote the clinical application of artificial intelligence-assisted technologies such as Fetal Intelligent Navigation Echocardiography (FINE), utilizing four-dimensional spatiotemporal image correlation to acquire fetal cardiac volume data and automatically identify and display standard views, thereby enhancing diagnostic reproducibility and efficiency (84). These innovations in imaging will provide FCI with more precise preoperative evaluation and postoperative follow-up, thereby optimizing clinical decision-making and improving patient management.

### Development of fetal-specific devices

Currently, the balloons, catheters, guidewires, and stents used in FCI are not specifically designed for the fetus, which, to some extent, limits procedural success and safety. One of the future directions for FCI is the development of interventional devices specifically designed for fetal use. Specific requirements include: (1) balloons and catheters with smaller diameters and increased flexibility to minimize myocardial injury; (2) low-profile stents with high radial strength, designed specifically for cases with thickened atrial septum, to reduce the risk of dislodgement (58); and (3) further optimizing the laser-assisted puncture systems to improve success rates in atrial septal puncture (42, 60). These device advancements will directly contribute to safer and more effective FCI procedures.

### Long-term follow-up outcomes

Current outcome studies in FCI primarily focus on technical success, live birth, and BVC rates, with insufficient attention given to long-term outcomes in survivors. This focus, combined with the absence of large, multicenter cohorts, has resulted in a lack of high-quality clinical evidence. Consequently, establishing standardized long-term follow-up systems is a critical future direction. Specific needs include: (1) evaluating neurodevelopment, cardiovascular function, exercise capacity, quality of life, and social integration in survivors of different FCI types during school age, adolescence, and adulthood (81); (2) promoting multicenter registries and collaborative studies to refine patient selection, optimize technical advancements, and determine the ideal timing for FCI; and (3) establishing matched comparisons with natural history cohorts to more accurately evaluate the true treatment effects of FCI (53).

### Ethical, psychosocial, and multidisciplinary considerations

FCI requires a careful balance between potential fetal benefit and maternal risk. While previous studies report minimal maternal complications associated with FCI in experienced centers (85, 86), long-term maternal outcomes still require further investigation. The process of prenatal CHD diagnosis and the consideration of FCI can lead to significant family distress, underscoring the importance of providing structured perinatal psychological support. Although academic organizations such as the Fetal Heart Society and existing guidelines (87–91) advocate for integrated care models, the development of high-level evidence-based guidelines remains challenging due to the high heterogeneity of CHD, significant variability in institutional treatment protocols, and insufficient systematic collaboration mechanisms (90). Studies by Wautlet et al. (92), Ronai et al. (93), and Zhang et al. (94) have demonstrated that structured multidisciplinary management strategies can improve clinical outcomes in children with various CHDs following FCI. The future direction lies in developing consensus-based standardized clinical pathways. Specific measures include: (1) defining the core composition of the FCI multidisciplinary team, including specialists in obstetrics, maternal-fetal medicine, pediatric cardiology, fetal surgery, cardiothoracic surgery, imaging, anesthesiology, palliative care, genetics, psychology, social work, and nursing (95); (2) establishing standardized operating protocols covering the entire process from prenatal diagnosis, preoperative evaluation, interventional procedure, delivery management, postnatal intervention, to long-term follow-up; and (3) evaluating the actual impact of standardized clinical pathways on reducing maternal-fetal risks, improving neonatal and long-term outcomes, and lowering healthcare costs (96).

## Conclusion

FCI has emerged as a transformative treatment for selected severe CHDs. The procedural indications, clinical outcomes, and associated complications of FCI are summarized in Table 2. But most studies included in this article had small sample sizes and were single-center retrospective designs. Therefore, the true efficacy of FCI in CHD should be interpreted with caution. Future collaborative, multi-center initiatives with standardized long-term follow-up protocols are needed to address these critical evidence gaps. Future advancements in this field are expected to benefit from innovations in fetal imaging, the development of fetal-specific devices, and the implementation of integrated prenatal-postnatal care pathways. Through ongoing research and technical refinement, FCI holds significant promise for further improving the prognosis of fetuses with severe congenital heart defects.

**Table 2:** Indications, outcomes, and complications of FCI.

Catheter-Base FCI	Indications	Short outcomes	Complications	Long-term outcomes
FAV	AS with HLHS	Technical success rate: 70%–94% (3, 17, 27, 28, 44–51)	Bradycardia, Pericardial effusion, Hemothorax, Aortic regurgitation, Left ventricular diastolic dysfunction, Left ventricular thrombus, Placental abruption, Balloon rupture, Preterm birth, Intrauterine death	Unknown
		Fetal mortality: 7%–33% (3, 17, 27, 28, 44–46, 49–52)		
		BVC rate: 40%–74% (3, 27, 28, 44–48, 50–52)		
		Live birth rate: 50%–93% (3, 27, 28, 44–46, 48, 50–52)		
FASI	HLHS with R/IAS	Technical success rate: 56%–90% (3, 17, 18, 55, 56, 58–60)	Bradycardia, Pericardial effusion, Hemothorax, Balloon rupture, Stent dislodgement/displacement, Preterm birth, Intrauterine death	Unknown
		Fetal mortality: 7%–83% (3, 17, 18, 47, 55–60)		
		Live birth rate: 75%–92% (3, 18, 47, 55)		
FPV	PA/IVS	Technical success rate: 69%–91% (3, 17, 23, 47, 49, 62)	Bradycardia, Pericardial effusion, Hemothorax, Pulmonary valve restenosis/atresia, Severe tricuspid regurgitation, Balloon rupture, Intrauterine death, Preterm birth	Unknown
		Fetal mortality: 7%–25% (3, 17, 23, 49)		
		BVC rate: 25%–77% (3, 23, 62, 64, 65)		
		Live birth rate: 64%–85% (3, 20, 23)		

AS, aortic stenosis; HLHS, hypoplastic left heart syndrome; R/IAS, restrictive or intact atrial septum; PA, pulmonary atresia; IVS, intact ventricular septum; BVC, biventricular circulation.
